# Proton pump inhibitors associated acute kidney injury and chronic kidney disease: data mining of US FDA adverse event reporting system

**DOI:** 10.1038/s41598-021-83099-y

**Published:** 2021-02-11

**Authors:** Bin Wu, Dan Li, Ting Xu, Min Luo, Zhiyao He, Yuwen Li

**Affiliations:** 1 Department of Pharmacy, West China Hospital, Sichuan University, 37 Guoxue Alley, Wuhou, Chengdu, 610041 Sichuan China; 2 West China School of Medicine, Sichuan University, Chengdu, 610041 Sichuan China; 3 West China School of Pharmacy, Sichuan University, Chengdu, 610041 Sichuan China

**Keywords:** Drug safety, Kidney diseases

## Abstract

Proton pump inhibitors (PPIs) were widely used. Observational studies suggested increasing risk of kidney injury in patients with PPIs treatment. We gathered six PPI regimens and adverse reports of acute kidney injury (AKI) and chronic kidney disease (CKD) based on US FDA Adverse Event Reporting System (FAERS) database from 2004 to 2019. We employed reporting odds ratio (ROR) to detect signals. Finally, we identified 3187 PPIs-associated AKI cases and 3457 PPIs-associated CKD cases. We detected significant signals between PPIs and AKI as well as CKD. The signal strength was stronger for CKD (ROR = 8.80, 95% CI 8.49–9.13) than AKI (ROR = 3.95, 95% CI 3.81–4.10), while dexlansoprazole performed stronger association for CKD (ROR = 34.94, 95% CI 30.89–39.53) and AKI (ROR = 8.18, 95% CI 7.04–9.51) than the other five PPIs. The median time from PPIs use to event occurrence was 23 days for AKI and 177 days for CKD. PPIs-associated AKI resulted larger proportion of death, life-threatening, hospitalization and disability events than PPIs-associated CKD. By mining the FAERS big data, we provided more information between PPIs use and the AKI and CKD events. PPIs rational use should be repeatedly stressed.

## Introduction

Proton pump inhibitors (PPIs) were widely used to treat peptic ulcer disease (PUD), gastroesophageal reflux disease (GERD), *Helicobacter pylori* infection, and to prevent side effects of glucocorticoids or non-steroidal anti-inflammatory drugs (NSAIDs)^1^. However, PPIs were overused by off label indication, excessive dosage and long-term treatment^2,3^.

With the widespread use of PPIs, more and more studies concerned for the safety of PPIs treatment^4–7^. Among which, kidney injury including acute kidney injury (AKI) and chronic kidney disease (CKD) following PPI therapy was a hot issue. However, original studies concerning PPIs-associated kidney injury were almost cohort or retrospective studies and systematic reviews based on them^8–11^, the only randomized controlled trial evaluated only pantoprazole and found no significant relationship between pantoprazole and CKD^12^.

Adverse event reporting system data was an outstanding source for post-marketing drug safety monitoring and pharmacovigilance analysis. The US food and drug administration (FDA) adverse event reporting system (FAERS) is one of the largest databases open to the public^13^. To the end of 2019, FAERS had collected more than ten million of cases, containing adverse drug event reports submitted by healthcare professionals, manufacturers, consumers and lawyers. These reports could be quantitatively analyzed using data mining methods to detect signals of drug-associated adverse events^14,15^. The objective of present study was to detect the signal of PPI-associated renal injury by systematically assessing spontaneous reports submitted to the FAERS database.

## Results

### Characteristics analysis

**Figure 1:**
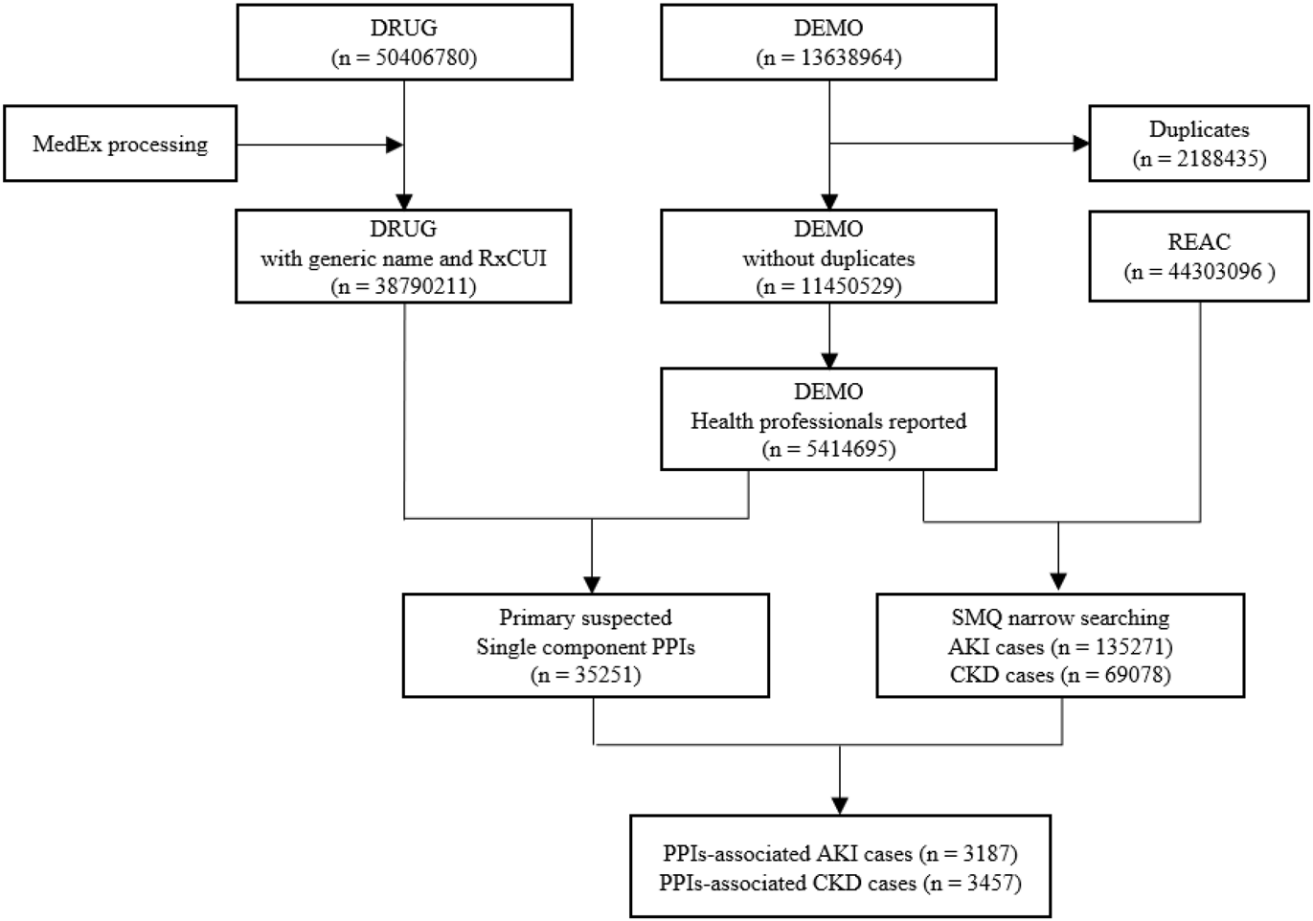
Flow chart of identifying PPI associated AKI and CKD cases from FAERS database.

After data cleaning, we retrieved a total of 11,450,529 cases from January 2004 to December 2019 from FAERS database, 5,414,695 of which were reported by health professionals (Fig. 1). We screened 35,251 PPIs-associated ADE cases reported by health professionals, including 10,299, 8963, 6093, 1439, 7273 and 1184 cases for omeprazole, pantoprazole, lansoprazole, rabeprazole, esomeprazole and dexlansoprazole, respectively. No case was identified for dexrabeprazole (ATC code: A02BC07). We further identified 3187 and 3457 PPIs users reported by health professionals with AKI and CKD events, respectively (Table 1). 1096 (34.39%) cases reported more than one kinds of PPIs in AKI group, however, the number was 1748 (51.61%) in CKD group. The concomitant drugs and adverse events were in Supplementary Table S1 and S2, respectively.

**Table 1:** Characteristics of PPIs associated AKI and CKD cases from FAERS database.

Characteristics	Subgroups	AKI		CKD	
		Cases/N	Proportion/%	Cases/N	Proportion/%
Cases	All PPIs	3187	100.00	3457	100.00
	Omeprazole	561	17.60	166	4.80
	Pantoprazole	999	31.30	1196	34.60
	Lansoprazole	1014	31.80	1542	44.60
	Rabeprazole	55	1.70	28	0.80
	Esomeprazole	353	11.10	158	4.60
	Dexlansoprazole	205	6.40	367	10.60
PPI	One kind	2091	65.61	1673	48.39
	Two or more kinds	1096	34.39	1784	51.61
Age	< 18 years	24	0.80	10	0.30
	18–65 years	752	23.60	559	16.20
	≥ 65 years	882	27.70	419	12.10
	Unknown	1529	48.00	2469	71.40
Sex	Female	1038	32.60	892	25.80
	Male	971	30.50	695	20.10
	Unknown	1178	37.00	1870	54.10
Reporter	Physician	665	20.90	314	9.10
	Other health professional	2255	70.80	3082	89.20
	Pharmacist	267	8.40	61	1.80
Country (top 5)	United States	1844	57.9	3015	87.20
	France	526	16.5	154	4.50
	Great Britain	289	9.10	61	1.80
	Japan	84	2.60	32	0.90
	Germany	59	1.90	34	1.00
Year	2019	1942	60.90	2851	82.50
	2018	391	12.30	231	6.70
	2017	140	4.40	45	1.30
	2016	207	6.50	35	1.00
	2004 to 2015	507	15.90	295	8.40

*PPIs* proton pump inhibitors, *AKI* acute kidney injury, *CKD* chronic kidney disease.

Among cases reported with age, the proportion of PPIs users in over-65-year group was larger than other age groups for AKI, while the proportion was larger in 18 to 65 group for CKD. Female cases were reported more than male, the female versus male ratio were 1.07 and 1.28 for the events of AKI and CKD, respectively.

Other health professional (70.80%) reported the most cases, followed by physician (20.90%) and pharmacist (8.40%). United States (57.90%) reported the most cases, followed by France (16.50%) and Great Britain (9.10%).

**Figure 2:**
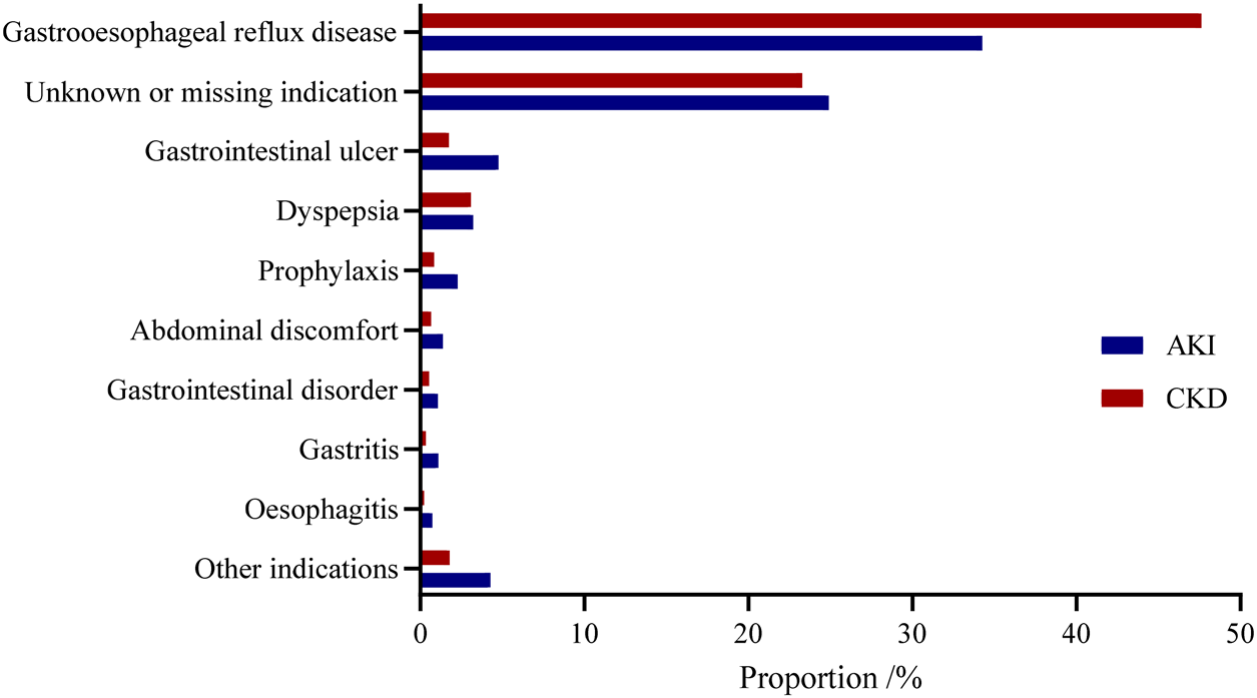
Indications of PPIs associated AKI and CKD cases from FAERS database. *PPIs* proton pump inhibitors, *AKI* acute kidney injury, *CKD* chronic kidney disease.

Gastroesophageal reflux disease was the most common indication, accounting for 41.21% (2738/6644) for all AKI and CKD cases. However, 24.07% (1599/6644) PPIs users were prescribed with unknown or missing indication. The other indications were shown in Fig. 2.

### Signal detection

We first conducted signal detection based on all PPIs, detected significant AKI and CKD signals. The strength of CKD signal (ROR = 8.80, 95% CI 8.49–9.13) was stronger than AKI (ROR = 3.95, 95% CI 3.81–4.10). Then we conducted signal detection in individual PPI, detected significant AKI and CKD signals in all the six PPIs (Table 2).

**Table 2:** Signal detection for AKI and CKD of PPIs users from FAERS database.

Renal injury	Drugs	Cases with renal injury/n	Cases with all AE/N	Proportion of renal injury cases in all AE cases/%	ROR	95% CI
AKI	All PPIs	3187	35,251	9.04	3.95	3.81–4.10
	Omeprazole	561	10,299	5.45	2.25	2.07–2.45
	Pantoprazole	999	8963	11.15	4.92	4.61–5.26
	Lansoprazole	1014	6093	16.64	7.84	7.33–8.39
	Rabeprazole	55	1439	3.82	1.55	1.18–2.03
	Esomeprazole	353	7273	4.85	1.99	1.79–2.22
	Dexlansoprazole	205	1184	17.31	8.18	7.04–9.51
CKD	All PPIs	3457	35,251	9.81	8.80	8.49–9.13
	Omeprazole	166	10,299	1.61	1.27	1.09–1.48
	Pantoprazole	1196	8963	13.34	12.11	11.39–12.87
	Lansoprazole	1542	6093	25.31	26.80	25.28–28.40
	Rabeprazole	28	1439	1.95	1.54	1.06–2.23
	Esomeprazole	158	7273	2.17	1.72	1.47–2.01
	Dexlansoprazole	367	1184	31.00	34.94	30.89–39.53

*PPIs* proton pump inhibitors, *AKI* acute kidney injury, *CKD* chronic kidney disease, *AE* adverse event, *ROR* reporting odds ratio, *95% CI* 95% confidence interval.

For AKI detection, dexlansoprazole detected the strongest signal (ROR = 8.18, 95% CI 7.04–9.51), followed by lansoprazole (ROR = 7.84, 95% CI 7.33–8.39), rabeprazole detected the weakest signal (ROR = 1.55, 95% CI 1.18–2.03).

For CKD detection, dexlansoprazole detected the strongest signal (ROR = 34.94, 95% CI 30.89–39.53), followed by lansoprazole (ROR = 26.80, 95% CI 25.28–28.40), omeprazole detected the weakest signal (ROR = 1.27, 95% CI 1.09–1.48).

### Time interval calculation

The time interval analysis included a total of 848 cases, including 562 cases for AKI and 286 cases for CKD.

**Figure 3:**
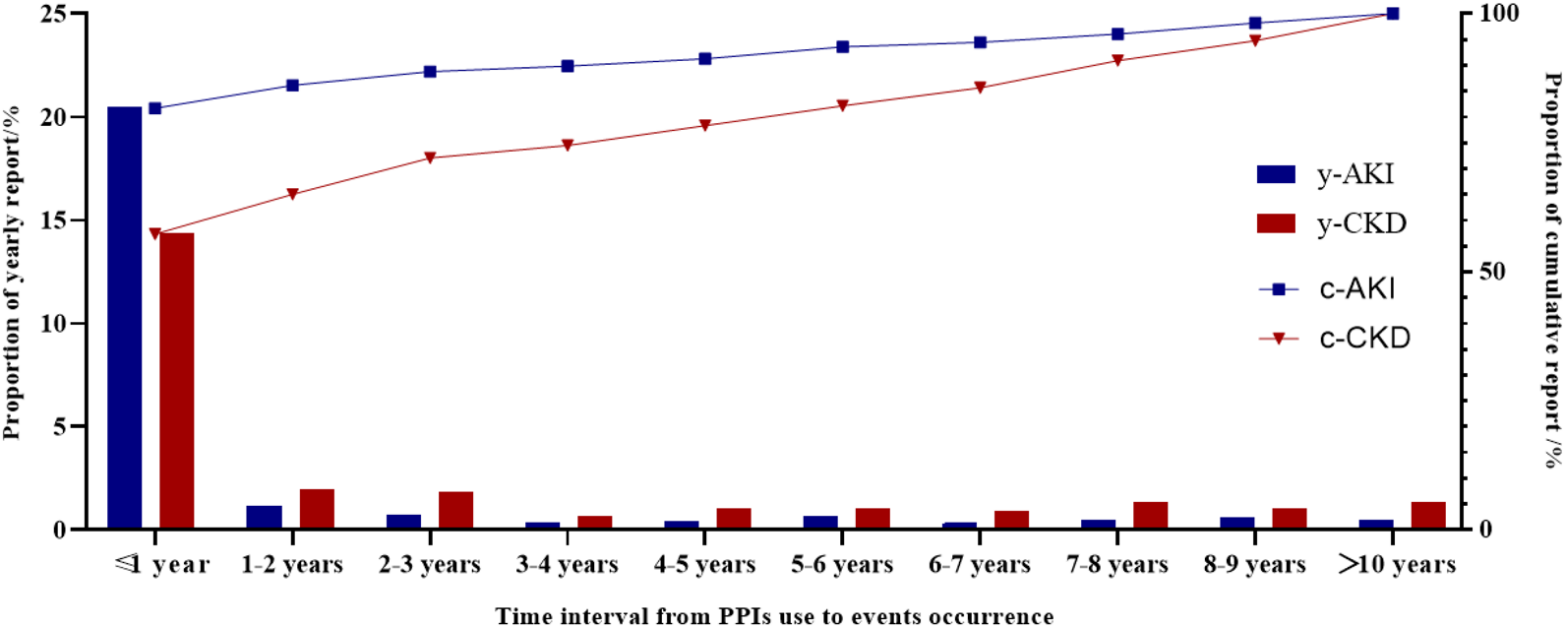
Time interval from PPIs use to AKI and CKD events occurrence from FAERS database. *PPIs* proton pump inhibitors, *AKI* acute kidney injury, *CKD* chronic kidney disease.

The biggest yearly reports proportion of PPIs-associated AKI and CKD events occurred in the first year (Fig. 3), accounting 81.67% (459/562) for AKI and 57.34% (164/286) CKD.

**Figure 4:**
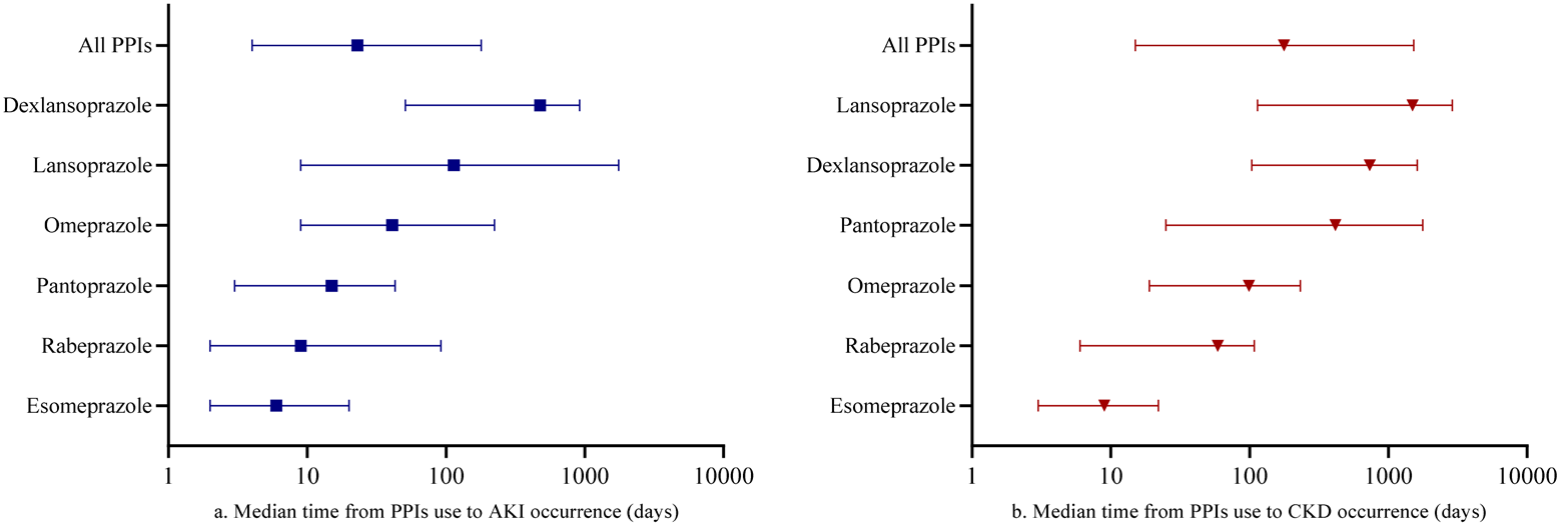
Median time from PPIs use to events occurrence from FAERS database. *PPIs* proton pump inhibitors, *AKI* acute kidney injury, *CKD* chronic kidney disease.

The median time from PPIs use to AKI occurrence was 23 (interquartile range (IQR) 4 to 179) days (Fig. 4a). The longest AKI occurrence median time was 476 (IQR 51 to 917) days for dexlansoprazole, while the shortest time was 6 (IQR 2 to 20) days for esomeprazole.

The median time from PPIs use to CKD occurrence was 177 (IQR 15 to 1528) days (Fig. 4b). The longest CKD occurrence median time was 1495 (IQR 114 to 2883) days for lansoprazole, while the shortest time was 9 (IQR 3 to 22) days for esomeprazole.

### Prognosis analysis

We analyzed outcomes of PPIs-associated AKI and CKD events. Compared with PPIs-associated CKD, PPIs-associated AKI resulted larger proportion of death, life-threatening, hospitalization and disability events. The individual PPI-associated AKI and CKD outcomes were shown in Table 3.

**Table 3:** Outcomes of cases reported as AKI and CDK using PPIs from FAERS database.

Renal injury	Drugs	Cases with renal injury/N	Death		Life-threatening		Hospitalization		Disability	
			Cases/n	Proportion/%	Cases/n	Proportion/%	Cases/n	Proportion/%	Cases/n	Proportion/%
AKI	All PPIs	3187	391	12.27	161	5.05	856	26.86	19	0.60
	Omeprazole	561	46	8.20	69	1.23	294	52.41	8	1.43
	Pantoprazole	999	111	11.11	29	0.29	245	24.52	7	0.70
	Lansoprazole	1014	150	14.79	14	0.14	93	9.17	2	0.20
	Rabeprazole	55	6	10.91	4	0.73	24	43.64	1	1.82
	Esomeprazole	353	57	16.15	44	1.25	195	55.24	1	0.28
	Dexlansoprazole	205	21	10.24	1	0.05	5	2.44	0	0.00
CKD	All PPIs	3457	375	10.85	65	0.19	296	8.56	16	0.46
	Omeprazole	166	19	11.45	32	1.93	76	45.78	7	4.22
	Pantoprazole	1196	122	10.20	10	0.08	99	8.28	7	0.59
	Lansoprazole	1542	174	11.28	4	0.03	30	1.95	1	0.06
	Rabeprazole	28	4	14.29	1	0.36	13	46.43	0	0.00
	Esomeprazole	158	24	15.19	17	1.08	73	46.20	1	0.63
	Dexlansoprazole	367	32	8.72	1	0.03	5	1.36	0	0.00

*PPIs* proton pump inhibitors, *AKI* acute kidney injury, *CKD* chronic kidney disease.

### Dosage analysis

The median daily dose (60 mg, IQR 60 to 60) of dexlansoprazole was the highest, twofold of the WHO DDD, followed by esomeprazole (CKD group), 1.33-fold of the WHO DDD, however the dose ranges were within that recommended by drug label. The median daily doses of other PPIs were equal to or lower than the WHO DDD (Table 4).

**Table 4:** Daily dose of PPIs from FAERS database.

Renal injury	Drugs	Cases with renal injury /N	Renal injury cases with daily dose reported /n	WHO DDD /mg	Daily dose recommended by drug label /mg	PPI daily dose /mg	
						Median	IQR
AKI	Omeprazole	561	217	20	20–60	20	20–40
	Pantoprazole	999	320	40	40	40	40–40
	Lansoprazole	1014	404	30	15–60	30	30–40
	Rabeprazole	55	6	20	20–60	20	10–20
	Esomeprazole	353	198	30	20–40	20	20–40
	Dexlansoprazole	205	56	30	30–60	60	60–60
CKD	Omeprazole	166	42	20	20–60	20	20–40
	Pantoprazole	1196	332	40	40	40	40–40
	Lansoprazole	1542	662	30	15–60	30	30–40
	Rabeprazole	28	3	20	20–60	20	10–20
	Esomeprazole	158	51	30	20–40	40	20–40
	Dexlansoprazole	367	107	30	30–60	60	60–60

*PPIs* proton pump inhibitors, *AKI* acute kidney injury, *CKD* chronic kidney disease, *DDD* defined daily dose IQR: interquartile range.

## Discussion

The present study investigated six PPIs-associated AKI and CKD, as well as indications and outcomes of reported cases, time interval to events occurrence and dosage of PPIs using. The results indicated significant association between AKI or CKD events and PPIs, including dexlansoprazole, lansoprazole, pantoprazole, omeprazole, esomeprazole and rabeprazole. The signal strength was stronger in CKD than AKI and varied across specific PPI regimens.

PPIs-associated renal injury had caught professionals’ attention, as well as the public and the media. Recently, many studies focused on PPIs associated AKI^8,16,17^ and CKD^9,18–21^. To reduce the influence of the public and the media, we had removed cases reported by consumers and lawyers. However, more than half the cases were reported in 2019, the risk of stimulated reporting could not be ruled out.

Overuse of PPIs was a worldwide problem. Our study found more than 20% PPI cases did not report clear indications. A PPI prescription survey of 45 hospitals published in 2019 indicated that between 32 and 56% of the PPI prescriptions did not have appropriate indications in China^2^. And an multicenter observational study in 2020 showed a range from 22 to 63% of PPIs treatment in Italian nursing homes were inappropriate^3^. To decrease drug related kidney injury, improper PPI use should be avoided firstly.

Omeprazole is the PPI with the longest history of clinical use and first report of PPIs-associated kidney injury^22^. However, the most cases reported of PPIs-associated AKI and CKD was lansoprazole, and the strongest signal of PPIs-associated AKI and CKD was dexlansoprazole from FAERS. Welch analyzed drug-associated AKI compared with all other drugs using FAERS data from 2004 to 2015, found the ROR was 2.35 (95% CI 2.19–2.53) for omeprazole^23^, being consistent with the result of our study. Chen detected PPIs-associated AKI based on cases reported by all reporters in FAERS from 2004 to 2019, the ROR value of each PPI was bigger than our study^17^, indicated over-estimation could be reduced to a certain extent by removing non-health professionals' reports.

Our study found the highest yearly proportion of PPIs-associated AKI and CKD was within the first years after PPIs treatment and the median time was 23 days for AKI and 177 days for CKD. Chen found a longer median time (446 days) for PPIs-associated AKI^17^. Studies revealed the risk of AKI or CKD increased with cumulative or prolonged PPIs use^20,24^. Unnecessary long-term PPIs use should be avoided.

Studies revealed PPIs was associated with increased risk of death^25–27^. Research reported the all cause death ratio of PPIs was 13.40%, 4861 deaths out of 36,282 PPIs users, and PPIs showed higher odds (OR 1.76) for mortality after logistic analysis^25^. Another study reported the estimated death ratio due to PPIs-induced CKD was 0.419%^26^. In our study, outcome analysis revealed all cause death proportion (PPIs-associated renal injury number as denominator) was 10.85% for PPIs-associated CKD and 12.27% for PPIs-associated AKI. Because the FAERS data did not include PPI users without adverse event occurrence, the incidence rate could not be calculated. However, such a high proportion of death and hospitalization could still attract our attention.

The findings of the present study and the existing evidence should arouse vigilance of PPI use. PPIs should be prescribed or sold following proven indication, low dose and short course^28^. PPIs users with no or unknown indication should stop PPI treatment, and those with indications should avoid long term PPI use if unnecessary^26^. Patients with primary kidney disease should pay more attention using PPIs. Monitoring of kidney function for long-term PPI users was necessary^26^.

Our study revealed significant association between AKI, CKD and PPIs treatment based on the FAERS real world big data, however, certain limitations existed. Like other spontaneous reporting system, FAERS is voluntary and opened to health professional as well as general public, so under-reporting, over-reporting, varied or missing information was inevitable^29^. Some calculation, especially PPIs duration and PPIs dose, just included cases with data reported. It was also difficult to perform risk factors analysis between PPIs and kidney injury for the limited information. Although non-health professionals’ reports excluded, the risk of stimulated reporting could not be eliminated.

## Conclusion

The present study identified six PPIs-associated AKI and CKD signals based on the FAERS data. Significant signal strength was stronger for CKD than AKI, while dexlansoprazole performed stronger association for AKI and CKD than the other five PPIs. A high proportion of renal injury occurred after the first year of PPI treatment and the median time was longer for CKD than AKI. Besides, more than 20% PPI treatment did not have clear indications. Our findings suggest PPIs-associated AKI and CKD should be taken into account, and the importance of PPIs rational use should be reemphasized.

## Methods

### Data source

We downloaded 64 quarters of adverse event report datasets from the FAERS Quarterly Data Extract Files website^30^, covering the period from January 2004 to December 2019.

Each quarterly data was consisted of seven data tables, including the DEMO table for patient demographic and administrative information, the DRUG table for the drug information, the REAC table for adverse events information, the OUTC table for patient outcomes information, the RPSR table for report sources information, the THER table for drug therapy start and end dates information and the INDI table for the indications for drug use. We imported all data into the local FAERS database, managed by Microsoft SQL server 2017 software.

We de-duplicated cases from the original datasets following the FDA recommendations. We removed the same records from the DEMO table and kept one, then removed the earliest FDA_DT when the CASEIDs were the same and removed the lower PRIMARYID when the CASEID and FDA_DT were the same. To make the evaluation more accurately, we only included cases reported by health professionals, including physician, pharmacist and other health professional in the OCCP_COD column of the DEMO table.

### PPIs identification

In the DRUG table, drugs could be documented in brand names, synonymous names or their abbreviations besides generic names in the DRUGNAME or the PROD_AI columns. We used the MedEx software (MedEx UIMA 1.3.7, Vanderbilt university, US) to transform different forms of drug name into the “generic name”, and add an identification code named RXNORM_RxCUI^31,32^.

We tried to identify the seven single component PPI regimens with the WHO Anatomical Therapeutic Chemical (ATC) code of A02BC from the local FAERS database. The seven PPIs included omeprazole (ATC code: A02BC01), pantoprazole (ATC code: A02BC02), lansoprazole (ATC code: A02BC03), rabeprazole (ATC code: A02BC04), esomeprazole (ATC code: A02BC05), dexlansoprazole (ATC code: A02BC06) and dexrabeprazole (ATC code: A02BC07). We restricted the drug role as primary suspected (PS) drug in the DRUG table.

### Events identification

According to the Medical Dictionary for Regularly Activities (MedDRA) and Standardised MedDRA Queries (SMQs) version 23.1. We identified AKI cases using SMQ coded 20000003 narrow searching in the REAC table, including 19 Preferred Terms (PTs). We then identified CKD cases using SMQ coded 20000213 narrow searching, including 38 PTs. One case could be reported more than one PTs of the same SMQ, duplicate records was removed. For example, one case reported two records of “acute kidney injury” and “acute phosphate nephropathy”, we counted the two records as one AKI case. The PTs details could be found in Supplementary Table S3.

### Data mining

We counted the characteristics of AKI and CKD cases with PPIs use, including cases attributed to different PPIs, age and sex of patient, occupation and country of reporter, year of event reported and indications for PPIs application.

We employed reporting odds ratio (ROR) to detect the signals of AKI and CKD relevant to PPIs. In current study, ROR was the degree of disproportionate reporting of AKI or CKD event for PPIs compared to the same event for all other drugs in the FAERS database. The calculation method of ROR, 95% confidence interval (95% CI) for ROR were shown in Table 5. A significant signal is detected when the report cases ≥ 3 and the lower limit of 95% CI exceed one^33^. Basically, the higher the ROR value, the stronger the strength of the signal^13^.

**Table 5:** Two-by-two contingency table for reporting odds ratio analyses.

Drugs	AKI/CKD cases	All other adverse event cases
PPIs	a	b
All other drugs	c	d
ROR = $\frac{\mathrm{a}/\mathrm{b}}{\mathrm{c}/\mathrm{d}}$, 95% CI for ROR = exp (ln (ROR) ± 1.96 $\sqrt{\frac{1}{\mathrm{a}}+\frac{1}{\mathrm{b}}+\frac{1}{\mathrm{c}}+\frac{1}{\mathrm{d}}}$)		

*PPIs* proton pump inhibitors, *AKI* acute kidney injury, *CKD* chronic kidney disease, *ROR* reporting odds ratio, *95% CI* 95% confidence interval.

We further estimated the time interval from PPIs use to AKI and CKD events occurrence. We unified the time format as yyyy-mm-dd. The time interval was calculated using event date (EVENT_DT) in the DEMO table minus drug start date (START_DT) in the THER table. To make the calculation more accurately, we excluded cases not in the period of 2004 to 2019, cases without year, month or day data in either EVENT_DT or START_DT field, and cases with earlier event date than drug start date.

We also analyzed patient outcomes for the events, including death, life-threatening, hospitalization and disability for the PPIs associated AKI and CKD cases. If a case reported more than one outcome, we kept the worst one, for instance, one case reported both death and life-threatening, we removed the life-threatening one.

Finally, we analyzed PPIs daily dose and compared to the defined daily dose (DDD) recommended by the world health organization (WHO) or the label dose.

The statistical analyses were conducted by SPSS version 20.0 (IBM corporation, Armonk, New York, USA) and GraphPad prism version 8.0.2 (GraphPad Software, San Diego, California, USA).

## Supplementary Information

Supplementary Information.
