# Supplementary material for: Proton pump inhibitors associated acute kidney injury and chronic kidney disease: data mining of US FDA adverse event reporting system
Source: Sci Rep. 2021 Feb 11;11:3690. doi: 10.1038/s41598-021-83099-y (PMC7878877; doi:10.1038/s41598-021-83099-y)
Supplement: Supplementary file 1 — Supplementary Information. [file 41598_2021_83099_MOESM1_ESM.zip › Supplementary Table S1-S3 R1/Supplementary Table S1-S3 R1.docx]

**Proton pump inhibitors associated acute kidney injury and chronic kidney disease: data mining of US FDA adverse event reporting system**

Bin Wu, Dan Li, Ting Xu*, Min Luo, Zhiyao He, Yuwen Li

**Supplementary Table S1. Top 50 concomitant drugs**

| **No.** | **ATC classification 1** | **ATC classification 2** | **ATC code** | **Concomitant drugs** |
| --- | --- | --- | --- | --- |
| 1 | A | A02 | A02BA02 | ranitidine |
| 2 | B | B01 | B01AC06 | aspirin |
| 3 | C | C03 | C03CA01 | furosemide |
| 4 | C | C10 | C10AA05 | atorvastatin |
| 5 | C | C09 | C09BB03 | lisinopril |
| 6 | N | N02 | N02BE01 | acetaminophen |
| 7 | A | A02 | A02BA03 | famotidine |
| 8 | C | C08 | C08CA01 | amlodipine |
| 9 | N | N02 | N02AX02 | tramadol |
| 10 | C | C07 | C07AB02 | metoprolol |
| 11 | H | H03 | H03AA01 | levothyroxine |
| 12 | C | C10 | C10AA01 | simvastatin |
| 13 | N | N03 | N03AX12 | gabapentin |
| 14 | M | M01 | M01AE01 | ibuprofen |
| 15 | A | A10 | A10BA02 | metformin |
| 16 | J | J01 | J01FA10 | azithromycin |
| 17 | J | J01 | J01CA04 | amoxicillin |
| 18 | C | C09 | C09CA01 | losartan |
| 19 | J | J01 | J01MA02 | ciprofloxacin |
| 20 | M | M04 | M04AA01 | allopurinol |
| 21 | B | B01 | B01AC04 | clopidogrel |
| 22 | H | H02 | H02AB07 | prednisone |
| 23 | C | C07 | C07AG02 | carvedilol |
| 24 | C | C10 | C10AA07 | rosuvastatin |
| 25 | J | J01 | J01MA12 | levofloxacin |
| 26 | A | A02 | A02BA01 | cimetidine |
| 27 | C | C03 | C03AA03 | hydrochlorothiazide |
| 28 | M | M01 | M01AE02 | naproxen |
| 29 | R | R03 | R03AC02 | salbutamol |
| 30 | N | N06 | N06AB04 | citalopram |
| 31 | C | C10 | C10AA03 | pravastatin |
| 32 | A | A04 | A04AA01 | ondansetron |
| 33 | A | A11 | A11CC01 | ergocalciferol |
| 34 | A | A02 | A02AC01 | calcium carbonate |
| 35 | A | A02 | A02BX05 | bismuth subcitrate |
| 36 | N | N05 | N05BA12 | alprazolam |
| 37 | A | A11 | A11CC05 | colecalciferol |
| 38 | C | C03 | C03DA01 | spironolactone |
| 39 | N | N02 | N02AA05 | oxycodone |
| 40 | M | M03 | M03BX08 | cyclobenzaprine |
| 41 | J | J01 | J01DB01 | cefalexin |
| 42 | N | N06 | N06AA09 | amitriptyline |
| 43 | C | C07 | C07AB03 | atenolol |
| 44 | J | J01 | J01AA02 | doxycycline |
| 45 | C | C02 | C02DB02 | hydralazine |
| 46 | A | A12 | A12BA01 | potassium chloride |
| 47 | C | C02 | C02AC01 | clonidine |
| 48 | C | C09 | C09CA03 | valsartan |
| 49 | R | R06 | R06AA02 | diphenhydramine |
| 50 | B | B01 | B01AA03 | warfarin |

**Supplementary Table S2. Top 10 concomitant adverse events of each SOC (n ＞= 10)**

| **SOC international number** | **System Organ Class (SOC)** | **No.** | **Preferred Term (PT)** |
| --- | --- | --- | --- |
| 1 | Blood and lymphatic system disorders | 1 | Anaemia |
| 1 | Blood and lymphatic system disorders | 2 | Thrombocytopenia |
| 1 | Blood and lymphatic system disorders | 3 | Eosinophilia |
| 1 | Blood and lymphatic system disorders | 4 | Pancytopenia |
| 1 | Blood and lymphatic system disorders | 5 | Neutropenia |
| 1 | Blood and lymphatic system disorders | 6 | Agranulocytosis |
| 1 | Blood and lymphatic system disorders | 7 | Leukocytosis |
| 1 | Blood and lymphatic system disorders | 8 | Disseminated intravascular coagulation |
| 1 | Blood and lymphatic system disorders | 9 | Bone marrow failure |
| 1 | Blood and lymphatic system disorders | 10 | Jaundice |
| 2 | Cardiac disorders | 1 | Dyspnoea |
| 2 | Cardiac disorders | 2 | Cardiac arrest |
| 2 | Cardiac disorders | 3 | Cardio-respiratory arrest |
| 2 | Cardiac disorders | 4 | Cardiac failure |
| 2 | Cardiac disorders | 5 | Coronary artery disease |
| 2 | Cardiac disorders | 6 | Oedema peripheral |
| 2 | Cardiac disorders | 7 | Cardiac failure congestive |
| 2 | Cardiac disorders | 8 | Dizziness |
| 2 | Cardiac disorders | 9 | Myocardial infarction |
| 2 | Cardiac disorders | 10 | Bradycardia |
| 5 | Endocrine disorders | 1 | Hypoglycaemia |
| 5 | Endocrine disorders | 2 | Diabetes mellitus |
| 7 | Gastrointestinal disorders | 1 | Diarrhoea |
| 7 | Gastrointestinal disorders | 2 | Vomiting |
| 7 | Gastrointestinal disorders | 3 | Nausea |
| 7 | Gastrointestinal disorders | 4 | Abdominal pain |
| 7 | Gastrointestinal disorders | 5 | Abdominal pain upper |
| 7 | Gastrointestinal disorders | 6 | Colitis microscopic |
| 7 | Gastrointestinal disorders | 7 | Colitis |
| 7 | Gastrointestinal disorders | 8 | Dyspepsia |
| 7 | Gastrointestinal disorders | 9 | Constipation |
| 7 | Gastrointestinal disorders | 10 | Gastric cancer |
| 8 | General disorders and administration site conditions | 1 | Death |
| 8 | General disorders and administration site conditions | 2 | Pyrexia |
| 8 | General disorders and administration site conditions | 3 | Fatigue |
| 8 | General disorders and administration site conditions | 4 | Decreased appetite |
| 8 | General disorders and administration site conditions | 5 | General physical health deterioration |
| 8 | General disorders and administration site conditions | 6 | Asthenia |
| 8 | General disorders and administration site conditions | 7 | Malaise |
| 8 | General disorders and administration site conditions | 8 | Rebound effect |
| 8 | General disorders and administration site conditions | 9 | Inflammation |
| 8 | General disorders and administration site conditions | 10 | Lethargy |
| 9 | Hepatobiliary disorders | 1 | Hepatocellular injury |
| 9 | Hepatobiliary disorders | 2 | Cholestasis |
| 9 | Hepatobiliary disorders | 3 | Hepatic failure |
| 9 | Hepatobiliary disorders | 4 | Hepatitis |
| 9 | Hepatobiliary disorders | 5 | Hepatic function abnormal |
| 9 | Hepatobiliary disorders | 6 | Hepatitis fulminant |
| 9 | Hepatobiliary disorders | 7 | Drug-induced liver injury |
| 9 | Hepatobiliary disorders | 8 | Hepatitis cholestatic |
| 9 | Hepatobiliary disorders | 9 | Hepatic encephalopathy |
| 9 | Hepatobiliary disorders | 10 | Cholangitis |
| 10 | Immune system disorders | 1 | Drug reaction with eosinophilia and systemic symptoms |
| 10 | Immune system disorders | 2 | Toxic epidermal necrolysis |
| 10 | Immune system disorders | 3 | Toxic skin eruption |
| 10 | Immune system disorders | 4 | Glomerulonephritis |
| 10 | Immune system disorders | 5 | Nephritis allergic |
| 10 | Immune system disorders | 6 | Hypersensitivity |
| 10 | Immune system disorders | 7 | Interstitial lung disease |
| 10 | Immune system disorders | 8 | Vasculitic rash |
| 11 | Infections and infestations | 1 | Sepsis |
| 11 | Infections and infestations | 2 | Septic shock |
| 11 | Infections and infestations | 3 | Urinary tract infection |
| 11 | Infections and infestations | 4 | Pneumonia |
| 11 | Infections and infestations | 5 | Candida infection |
| 11 | Infections and infestations | 6 | Upper respiratory tract infection |
| 12 | Injury, poisoning and procedural complications | 1 | Renal injury |
| 12 | Injury, poisoning and procedural complications | 2 | Toxicity to various agents |
| 12 | Injury, poisoning and procedural complications | 3 | Fall |
| 12 | Injury, poisoning and procedural complications | 4 | Overdose |
| 12 | Injury, poisoning and procedural complications | 5 | Off label use |
| 12 | Injury, poisoning and procedural complications | 6 | Intentional overdose |
| 12 | Injury, poisoning and procedural complications | 7 | Vasoplegia syndrome |
| 12 | Injury, poisoning and procedural complications | 8 | Post procedural complication |
| 13 | Investigations | 1 | Blood creatinine increased |
| 13 | Investigations | 2 | Weight decreased |
| 13 | Investigations | 3 | Haemoglobin decreased |
| 13 | Investigations | 4 | Hepatic enzyme increased |
| 13 | Investigations | 5 | Glomerular filtration rate decreased |
| 13 | Investigations | 6 | Blood pressure decreased |
| 13 | Investigations | 7 | C-reactive protein increased |
| 13 | Investigations | 8 | Electrocardiogram QT prolonged |
| 13 | Investigations | 9 | Blood bilirubin increased |
| 13 | Investigations | 10 | International normalised ratio increased |
| 14 | Metabolism and nutrition disorders | 1 | Hyperkalaemia |
| 14 | Metabolism and nutrition disorders | 2 | Dehydration |
| 14 | Metabolism and nutrition disorders | 3 | Lactic acidosis |
| 14 | Metabolism and nutrition disorders | 4 | Metabolic acidosis |
| 14 | Metabolism and nutrition disorders | 5 | Hyponatraemia |
| 14 | Metabolism and nutrition disorders | 6 | Hypomagnesaemia |
| 14 | Metabolism and nutrition disorders | 7 | Hypokalaemia |
| 14 | Metabolism and nutrition disorders | 8 | Hypocalcaemia |
| 14 | Metabolism and nutrition disorders | 9 | Respiratory failure |
| 14 | Metabolism and nutrition disorders | 10 | Acidosis |
| 15 | Musculoskeletal and connective tissue disorders | 1 | Rhabdomyolysis |
| 15 | Musculoskeletal and connective tissue disorders | 2 | Back pain |
| 15 | Musculoskeletal and connective tissue disorders | 3 | Myalgia |
| 16 | Neoplasms benign, malignant and unspecified (incl cysts and polyps) | 1 | Renal haemangioma |
| 16 | Neoplasms benign, malignant and unspecified (incl cysts and polyps) | 2 | Neoplasm malignant |
| 17 | Nervous system disorders | 1 | Confusional state |
| 17 | Nervous system disorders | 2 | Loss of consciousness |
| 17 | Nervous system disorders | 3 | Headache |
| 17 | Nervous system disorders | 4 | Cerebrovascular accident |
| 17 | Nervous system disorders | 5 | Disorientation |
| 17 | Nervous system disorders | 6 | Seizure |
| 17 | Nervous system disorders | 7 | Agitation |
| 17 | Nervous system disorders | 8 | Somnolence |
| 17 | Nervous system disorders | 9 | Depressed level of consciousness |
| 17 | Nervous system disorders | 10 | Coma |
| 19 | Psychiatric disorders | 1 | Depression |
| 19 | Psychiatric disorders | 2 | Anxiety |
| 19 | Psychiatric disorders | 3 | Suicide attempt |
| 20 | Renal and urinary disorders | 1 | Tubulointerstitial nephritis |
| 20 | Renal and urinary disorders | 2 | Nephropathy |
| 20 | Renal and urinary disorders | 3 | Renal tubular necrosis |
| 20 | Renal and urinary disorders | 4 | Renal disorder |
| 20 | Renal and urinary disorders | 5 | Proteinuria |
| 20 | Renal and urinary disorders | 6 | Haematuria |
| 20 | Renal and urinary disorders | 7 | Urinary retention |
| 20 | Renal and urinary disorders | 8 | Leukocyturia |
| 20 | Renal and urinary disorders | 9 | Nephrolithiasis |
| 20 | Renal and urinary disorders | 10 | Renal tubular disorder |
| 22 | Respiratory, thoracic and mediastinal disorders | 1 | Epistaxis |
| 22 | Respiratory, thoracic and mediastinal disorders | 2 | Pneumonia aspiration |
| 22 | Respiratory, thoracic and mediastinal disorders | 3 | Cough |
| 22 | Respiratory, thoracic and mediastinal disorders | 4 | Hypoxia |
| 22 | Respiratory, thoracic and mediastinal disorders | 5 | Chronic obstructive pulmonary disease |
| 22 | Respiratory, thoracic and mediastinal disorders | 6 | Pleural effusion |
| 22 | Respiratory, thoracic and mediastinal disorders | 7 | Acute respiratory distress syndrome |
| 22 | Respiratory, thoracic and mediastinal disorders | 8 | Respiratory distress |
| 22 | Respiratory, thoracic and mediastinal disorders | 9 | Respiratory arrest |
| 23 | Skin and subcutaneous tissue disorders | 1 | Rash |
| 23 | Skin and subcutaneous tissue disorders | 2 | Rash maculo-papular |
| 23 | Skin and subcutaneous tissue disorders | 3 | Pruritus |
| 25 | Surgical and medical procedures | 1 | Therapy cessation |
| 26 | Vascular disorders | 1 | Hypotension |
| 26 | Vascular disorders | 2 | Hypertension |
| 26 | Vascular disorders | 3 | Hypovolaemic shock |
| 26 | Vascular disorders | 4 | Circulatory collapse |
| 26 | Vascular disorders | 5 | Arteriosclerosis |
| 26 | Vascular disorders | 6 | Haemodynamic instability |
| 26 | Vascular disorders | 7 | Shock |

**Supplementary Table S3. SMQ narrow searching for AKI and CKD.**

| Renal injury | SMQ code | Preferred term code | Preferred terms |
| --- | --- | --- | --- |
| AKI | 20000003 | 10069339 | Acute kidney injury |
| AKI | 20000003 | 10069688 | Acute phosphate nephropathy |
| AKI | 20000003 | 10002847 | Anuria |
| AKI | 20000003 | 10003885 | Azotaemia |
| AKI | 20000003 | 10066338 | Continuous haemodiafiltration |
| AKI | 20000003 | 10061105 | Dialysis |
| AKI | 20000003 | 10078987 | Foetal renal impairment |
| AKI | 20000003 | 10018875 | Haemodialysis |
| AKI | 20000003 | 10053090 | Haemofiltration |
| AKI | 20000003 | 10049778 | Neonatal anuria |
| AKI | 20000003 | 10029155 | Nephropathy toxic |
| AKI | 20000003 | 10030302 | Oliguria |
| AKI | 20000003 | 10034660 | Peritoneal dialysis |
| AKI | 20000003 | 10072370 | Prerenal failure |
| AKI | 20000003 | 10038435 | Renal failure |
| AKI | 20000003 | 10038447 | Renal failure neonatal |
| AKI | 20000003 | 10062237 | Renal impairment |
| AKI | 20000003 | 10049776 | Renal impairment neonatal |
| AKI | 20000003 | 10081980 | Subacute kidney injury |
| CKD | 20000213 | 10053699 | Artificial kidney device user |
| CKD | 20000213 | 10003885 | Azotaemia |
| CKD | 20000213 | 10064848 | Chronic kidney disease |
| CKD | 20000213 | 10078095 | Chronic kidney disease-mineral and bone disorder |
| CKD | 20000213 | 10010082 | Coma uraemic |
| CKD | 20000213 | 10012660 | Diabetic end stage renal disease |
| CKD | 20000213 | 10061105 | Dialysis |
| CKD | 20000213 | 10059015 | Dialysis device insertion |
| CKD | 20000213 | 10077512 | End stage renal disease |
| CKD | 20000213 | 10018367 | Glomerulonephritis chronic |
| CKD | 20000213 | 10018875 | Haemodialysis |
| CKD | 20000213 | 10053090 | Haemofiltration |
| CKD | 20000213 | 10019845 | Hepatorenal failure |
| CKD | 20000213 | 10062624 | High turnover osteopathy |
| CKD | 20000213 | 10020708 | Hyperparathyroidism secondary |
| CKD | 20000213 | 10023421 | Kidney fibrosis |
| CKD | 20000213 | 10063000 | Low turnover osteopathy |
| CKD | 20000213 | 10058116 | Nephrogenic anaemia |
| CKD | 20000213 | 10067467 | Nephrogenic systemic fibrosis |
| CKD | 20000213 | 10029159 | Nephrosclerosis |
| CKD | 20000213 | 10049630 | Oedema due to renal disease |
| CKD | 20000213 | 10034498 | Pericarditis uraemic |
| CKD | 20000213 | 10034660 | Peritoneal dialysis |
| CKD | 20000213 | 10052279 | Renal and liver transplant |
| CKD | 20000213 | 10052278 | Renal and pancreas transplant |
| CKD | 20000213 | 10038435 | Renal failure |
| CKD | 20000213 | 10074746 | Renal replacement therapy |
| CKD | 20000213 | 10038519 | Renal rickets |
| CKD | 20000213 | 10038533 | Renal transplant |
| CKD | 20000213 | 10056609 | Uraemia odour |
| CKD | 20000213 | 10046324 | Uraemic acidosis |
| CKD | 20000213 | 10046326 | Uraemic encephalopathy |
| CKD | 20000213 | 10063709 | Uraemic gastropathy |
| CKD | 20000213 | 10077910 | Uraemic myopathy |
| CKD | 20000213 | 10046328 | Uraemic neuropathy |
| CKD | 20000213 | 10060875 | Uraemic pruritus |
| CKD | 20000213 | 10067863 | Uridrosis |
| CKD | 20000213 | 10081588 | Metabolic nephropathy |
